# Network pharmacology-based mechanism analysis of dauricine on the alleviating Aβ-induced neurotoxicity in *Caenorhabditis elegans*

**DOI:** 10.1186/s12906-024-04589-w

**Published:** 2024-08-30

**Authors:** Ranran Zhang, Xiaoyan Huang, Chunling Zhou, Qian Zhang, Dongsheng Jia, Xiaoliang Xie, Ju Zhang

**Affiliations:** 1 Institute of Cash Crops, Hebei Academy of Agriculture and Forestry Sciences, Shijiazhuang, China; 2 College of Bioscience and Engineering, Hebei University of Economics and Business, Shijiazhuang, China

**Keywords:** Dauricine, Alzheimer’s disease, Network pharmacology, *Caenorhabditis elegans*, Autophagy-lysosome

## Abstract

**Background:**

Dauricine (DAU), a benzyl tetrahydroisoquinoline alkaloid isolated from the root of *Menispermum dauricum* DC, exhibits promising anti-Alzheimer’s disease (AD) effects, but its underlying mechanisms remain inadequately investigated. This paper aims to identify potential targets and molecular mechanisms of DAU in AD treatment.

**Methods:**

Network pharmacology and molecular docking simulation method were used to screen and focus core targets. Various transgenic *Caenorhabditis elegans* models were chosen to validate the anti-AD efficacy and mechanism of DAU.

**Results:**

There are 66 potential DAU-AD target intersections identified from 100 DAU and 3036 AD-related targets. Subsequent protein-protein interaction (PPI) network analysis identified 16 core targets of DAU for anti-AD. PIK3CA, AKT1 and mTOR were predicted to be the central targets with the best connectivity through the analysis of “compound-target-biological process-pathway network”. Molecular docking revealed strong binding affinities between DAU and PIK3CA, AKT1, and mTOR. In vivo experiments demonstrated that DAU effectively reduced paralysis in AD nematodes caused by Aβ aggregation toxicity, downregulated expression of PIK3CA, AKT1, and mTOR homologues (*age-1*, *akt-1*, *let-363*), and upregulated expression of autophagy genes and the marker protein LGG-1. Simultaneously, DAU increased lysosomal content and enhanced degradation of the autophagy-related substrate protein P62. Thioflavin T（Th-T）staining experiment revealed that DAU decreased Aβ accumulation in AD nematodes. Further experiments also confirmed DAU’s protein scavenging activity in polyglutamine (polyQ) aggregation nematodes.

**Conclusion:**

Collectively, the mechanism of DAU against AD may be related to the activation of the autophagy-lysosomal protein clearance pathway, which contributes to the decrease of Aβ aggregation and the restoration of protein homeostasis.

**Supplementary Information:**

The online version contains supplementary material available at 10.1186/s12906-024-04589-w.

## Introduction

Alzheimer’s disease (AD) is a neurodegenerative disorder affecting the central nervous system, clinically characterized by memory loss, social impairment, behavioral difficulties and other symptoms. The global population increase and aging demographics have led to a continuous rise number and proportion of AD cases, posing significant social and economic burdens [1]. The multifactorial nature and complexity of AD’s etiology are revealed by several hypotheses about its pathogenesis [2–4]. The primary pathological hallmark of AD is the abnormal aggregation of amyloid beta (Aβ) protein in the brain. This aggregation disrupts the proteostasis, triggers neurotoxicity, and ultimately neuronal damage or death [5]. Therefore, reducing the pathological aggregation of Aβ proteins and restoring protein homeostasis are effective therapeutic strategies for AD. Currently approved anti-AD drugs, such as donepezil, carboplatin and galantamine, only alleviate AD symptoms without providing a radical cure. Moreover, they have considerable side effects and target a single aspect of drug action [6]. In recent years, traditional Chinese medicine ingredients have gained attention in anti-AD drug research due to their low side effects, affordability, and good basis for treating age-related neurodegenerative diseases.

Dauricine (DAU), a benzyl tetrahydroisoquinoline alkaloid extracted from the root of *Menispermum dauricum* DC, has diverse pharmacological activities, including anti-arrhythmic, anti-inflammatory, neuroprotective, anticancer and antioxidant effects [7–10]. DAU also shows promising therapeutic potential in AD. Chen et al. [11], demonstrated that DAU treatment in AD transgenic mice reduced abnormal Aβ aggregation and increased hippocampal ATP levels, resulting in significant improvement in cognitive dysfunction. Additionally, structurally modified oxoisoaporphine alkaloid derivatives extracted from the root of *Menispermum dauricum* DC also exhibit anti-AD effects by reducing Aβ1–42 secretion and decreasing its aggregation toxicity [12]. The collapse of proteostasis network is a hallmark feature of neurodegenerative disorders, thus the restoration of protein homeostasis represents a critical therapeutic strategy against AD [13, 14]. DAU exhibits anti-AD activity by activating endoplasmic reticulum-related pathways and unfolded protein responses, thereby reducing Aβ secretion and restoring protein homeostasis [15]. However, relevant studies on the anti-AD effect of DAU are still insufficient, and its potential targets and pharmacological activity mechanism need to be further explored.

Traditional “disease-target-drug” approaches often fail to capture the complex interplay between drug effects and disease mechanisms. Network pharmacology, which integrates bioinformatics and network analysis, offers a powerful tool for predicting drug-disease interactions and potential mechanisms of action. This methodology has been successfully applied to elucidate the potential targets and mechanisms of action of other drugs against AD, such as curcumin and cordycepin [16, 17]. *Caenorhabditis elegans* have been used as a well-established model organism for neurodegenerative disease research due to its ease of maintenance and culture, short life cycle, complete genome sequencing, and availability of various transgenic models associated with neurodegeneration [18].

Therefore, this study employed network pharmacology to predict potential DAU targets against AD, followed by in vivo functional validation in relevant *C. elegans* models. This combined approach aimed to investigate the potential targets and mechanisms of DAU against AD and provide theoretical and data support for its further development and therapeutic application.

## Materials and methods

### Animals

*C. elegans* strains N2 (wild type), CL2006 (dvIs2 [pCL12(unc-54/human Abeta peptide 1–42 minigene) + pRF4(rol-6(su1006))]), CL4176 (dvIs27 [pAF29(myo-3p::A-Beta 1–42::let-8513’UTR) + pRF4(rol-6(su1006))]X), DA2123 (adIs2122 [lgg-1p::GFP::lgg-1 + rol-6(su1006)]), BC12921 (sIs10729 [rCesT12G3.1::GFP + pCeh361]), CL2355 (dvIs50 [pCL45 (snb-1::Abeta 1–42::3’ UTR(long) + mtl-2::GFP]) and AM141 (rmIs133 [unc-54p::Q40::YFP])), and *Escherichia coli* strains (OP50 and NA22) were obtained from the Caenorhabditis Genetics Center (University of Minnesota, Minneapolis, MN, USA). Synchronized L1 larvae were prepared using the alkaline hypochlorite method.

### Network pharmacological analysis

DAU’s SMILES string structure from PubChem was imported into SwissADME to predict potential targets. AD-related targets were filtered from GeneCards using “Alzheimer’s disease” query with a relevance core > 10. Common targets were identified using Venny 2.1.0. Protein–protein interaction (PPI) networks were constructed using STRING database. Core targets were selected using Cytoscape v3.8.2 (Between Centrality > 0.01, Close Centrality > 0.45, and Degree > 8). Gene Ontology (GO) and Kyoto Encyclopedia of Genes and Genomes (KEGG) enrichment analyses were performed using DAVID (*P* < 0.05), visualized using Rstudio 1.4.1106.

### Molecular docking

DAU’s 3D structure was retrieved from PubChem and core target protein structures from UniProt/Protein Data Bank (PDB). After processed using AutoDock Tools-1.5.6, docking simulations were performed using PyRx-Virtual Screening Tool, visualized using Discovery Studio v16.1.0.15350.

### Food clearance assay

Synchronized L1 larvae nematodes were exposed to various concentration of DAU (Manstead Biotech, Chengdu, China) in S medium with *Escherichia coli (E. coli)* NA22 at the initial OD_570 nm_≈0.80 (96-well plate, 10–15/well, 10 wells/concentration), and then cultured at 20°C for assay. *E. coli* reduction rate was measured daily for 7 days using a full-wavelength microplate reader (BioTek, Vermont, USA) to determine safe DAU concentrations [19].

### AD nematode paralysis assay

DAU’s anti-paralytic effect was evaluated in CL4176/CL2006 strains expressing human Aβ protein in muscle. Synchronized L1 larvae nematodes were transferred onto nematode growth medium (NGM) plates (~ 40/plate) with OP50 and varying concentrations of DAU (3 replicates). CL4176 was incubated firstly at 15°C for 36 h and then shifted to 23°C (designated as 0 h) for paralysis assay at 2 h intervals until worms all became paralyzed. CL2006 was incubated at 20°C and added with 5-fluoro-2’-deoxyuridine (5-FuDR; Sigma, St. Louis, USA) for inhibition of spawning (designated as day 0) at L4 larvae. Paralysis rate of CL2006 was assessed every 2 days until completion. Worms that only moved their head but failed to move their body were scored as “paralyzed”.

### Real-time fluorescence quantitative PCR

Synchronized L1 nematodes (~ 1500) were cultured with/without DAU treatment for 3 days under the corresponding conditions until adulthood. Nematodes were collected, washed by M9 buffer, and RNA was extracted using TRIzol (Invitrogen, Carlsbad, USA). cDNA was quantified using SYBR Green (Toyobo, Osaka, Japan) in iQ5 real-time PCR System (Bio-Rad, Hercules, USA) [20]. Primers are listed in Table 1.

**Table 1:** Primer sequences used for real-time PCR

Genes	Forward primers	Reverse primers
*β-actin*	5’-TGCAGAAGGAAATCACCGCT-3’	5’-AGAAAGCTGGTGGTGACGAT-3’
*akt-1*	5’-GCCTAAGGAAGGACAACCG-3’	5’-ATGAATCCAACGCTGACGA-3’
*let-363*	5’-ACGTCAGAATATGGCTGGTG-3’	5’-AAGTGGTTCAGGTGGAGTTG-3’
*age-1*	5’-TGTGGGGACACTGACGCTG-3’	5’-TTGGCAGTCGGTTCAGGAG-3’
*bec-1*	5’-TGTTGAAAGAGCTCAAGGATCG-3’	5’-GGGGAAAAGGCAGAATTCCAG-3’
*epg-8*	5’-GCGGTAAACGCTACACAAAGA-3’	5’-CCATCCGCTGAGATTCCTGG-3’
*lgg-1*	5’-CGTGCCGAAGGAGACAAGAT-3’	5’-CTTCCTCGTGATGGTCCTGG-3’
*atg-18*	5’-AAGTTGGGGAGCTGATGACG-3’	5’-TGGTCTAAACGGATATGCTTGCT-3’
*unc-51*	5’-ATGAGAATGAGCCGTTGGAT-3’	5’-CGACTGAGGAGGAGGTTGAG-3’

### Autophagy activity assay

Autophagy was evaluated in DA2123 (expressing GFP::LGG-1) and BC12921 (expressing GFP-tagged P62). Synchronized L1 larvae nematodes were incubated at 20 °C for 72 h. Worms were paralyzed with isoflurane and analyzed by fluorescence microscopy (OPTIKA S. R. L, Bergamo, Italy; 30 worms/treatment for analysis). GFP-positive punctae were manually counted for DA2123, while fluorescence intensity was measured using the Thermo fluorescence microplate reader (excitation 485 nm, emission 535 nm) for BC12921 (20–30 nematodes/group, 3 replicates).

### Lysosome content assay

Lysosome content in CL4176 worms was quantified using a modified protocol [21]. Synchronized L1 larvae nematodes cultured with/without DAU (~ 700/well) were incubated at 15 °C (36 h) then 23 °C (24 h), and stained with 15 µM Lyso-Tracker red (Beyotime Biotechnology, Nanjing, China) for 1 h. Then nematodes were washed and imaged by fluorescence microscopy (10–20 nematodes/group, 3 replicates).

### Pharyngeal pumping assay

Synchronized L1 larvae N2 nematodes (~ 500) were cultured on NGM plates with/without DAU treatment until adulthood (20 °C, 72 h). Then nematodes were transferred to the fresh plates and counted the number of pharynx contraction individually for 30 s, using a stereomicroscope (30 worms/treatment for analysis, 3 replicates) [22].

### Oil Red O staining assay

Synchronized L1 larvae N2 nematodes (~ 1000) were cultured on NGM plate with/without DAU treatment at 20 °C for 3 days. Nematodes were collected and washed by M9 buffer three times. Then nematodes were immobilized with 80 µL of 4% paraformaldehyde for 30 min, and subsequently frozen at -80 °C to thaw and refroze for three times. After washing by M9 buffer, worms were stained with 120 µL of Oil Red O (ORO) working solution (Solarbio, Beijing, China) for 5 h at room temperature. Then after repeated washing, nematodes were imaged by a stereomicroscope outfitted with a color camera (OPTIKA S. R. L, Bergamo, Italy; 30 worms/treatment for analysis, 3 replicates independently) [23].

### Aβ aggregation assay

Synchronized L1 stage CL4176/CL2006 nematodes (~ 700/strain) treated with/without DAU were incubated at specific temperatures (CL4176: 15 °C/36 h, then 23 °C/36 h; CL2006: 20 °C/72 h). Following washes and fixation (4% polymethylal solution, 24 h), they were permeabilized (5% β-mercaptoethanol, 1% Triton, and 125 mM Tris-HCl at pH = 7.4; 37 °C/24 h) and stained with 0.125% Thioflavin T (Th-T) dye (Solarbio, Beijing, China) [24]. Anterior pharynx Aβ aggregation was imaged (20 nematodes/treatment, 3 replicates) by fluorescence microscopy.

### Chemotaxis assay

Chemotaxis assay was performed in CL2355 (Pan-neuronal expresion of Aβ) and CL2122 (control of CL2355, no Aβ) nematodes as previously described [19] with minor modifications. Synchronized L1 larvae nematodess (~ 700) were cultured firstly at 15 °C for 36 h, and then shifted to 23 °C for 36 h to induce Aβ expression. DAU administration was started differently at L1 larvae (before Aβ expression), and 12 h/ 24 h after Aβ induction. Nematodes were collected simultaneously 36 h after 23 °C induction and washed with M9 buffer 3 times. Then worms were transferred onto the center line of the 9 cm chemotaxis agar plate which was divided into normal (N) and trap (T) zones. After dropping the odorant attractant (0.1% benzaldehyde) and ethanol (control odorant) to the T and N zones, plates were covered and incubated at 23 °C for 60 min. Then the number of nematodes on the two zones was scored. Chemotaxis index was calculated as (T-N)/(T + N), where N and T represented the number of nematodes on the two (N/T) zones.

### PolyQ aggregation and body bends assay

PolyQ aggregation was assessed in AM141 nematodes, a Huntington’s disease (HD) model [20]. Synchronized L1 nematodes (~ 1000) were cultured with/without DAU at 20 °C. Fluorescent dots representing aggregates were quantified at 48, 72, and 96 h post DAU treatment by fluorescence microscopy (~ 30 nematodes/treatment, 3 replicates). The rate of body bends was measured at 72 and 96 h after DAU treatment (settling: 30 s, observation: 60 s, 20 nematodes/treatment, 3 replicates) [25]. A body bend was defined as a change in the direction of bending at the midbody.

### Statistical analysis

Statistical analysis was performed using GraphPad Prism 8.0 (GraphPad Software, San Diego, CA, USA). Image J software (V1.8.0.112) was applied to density quantification and fluorescence intensity analyze. Student’s t test or one-way ANOVA followed by Tukey’s post hoc test were used for determing the statistically significant differences between groups, and a *P* value < 0.05 was considered significant. Paralysis assay was analysed by Kaplan–Meier Survival Curves and calculated using the log-rank test for *P* value. All experiments were biologically replicated at least three times, and all values were presented as means ± SD or the representative.

## Results

### Network pharmacology reveals potential targets and pathways associated with DAU’s anti-AD activity

**Fig. 1:**
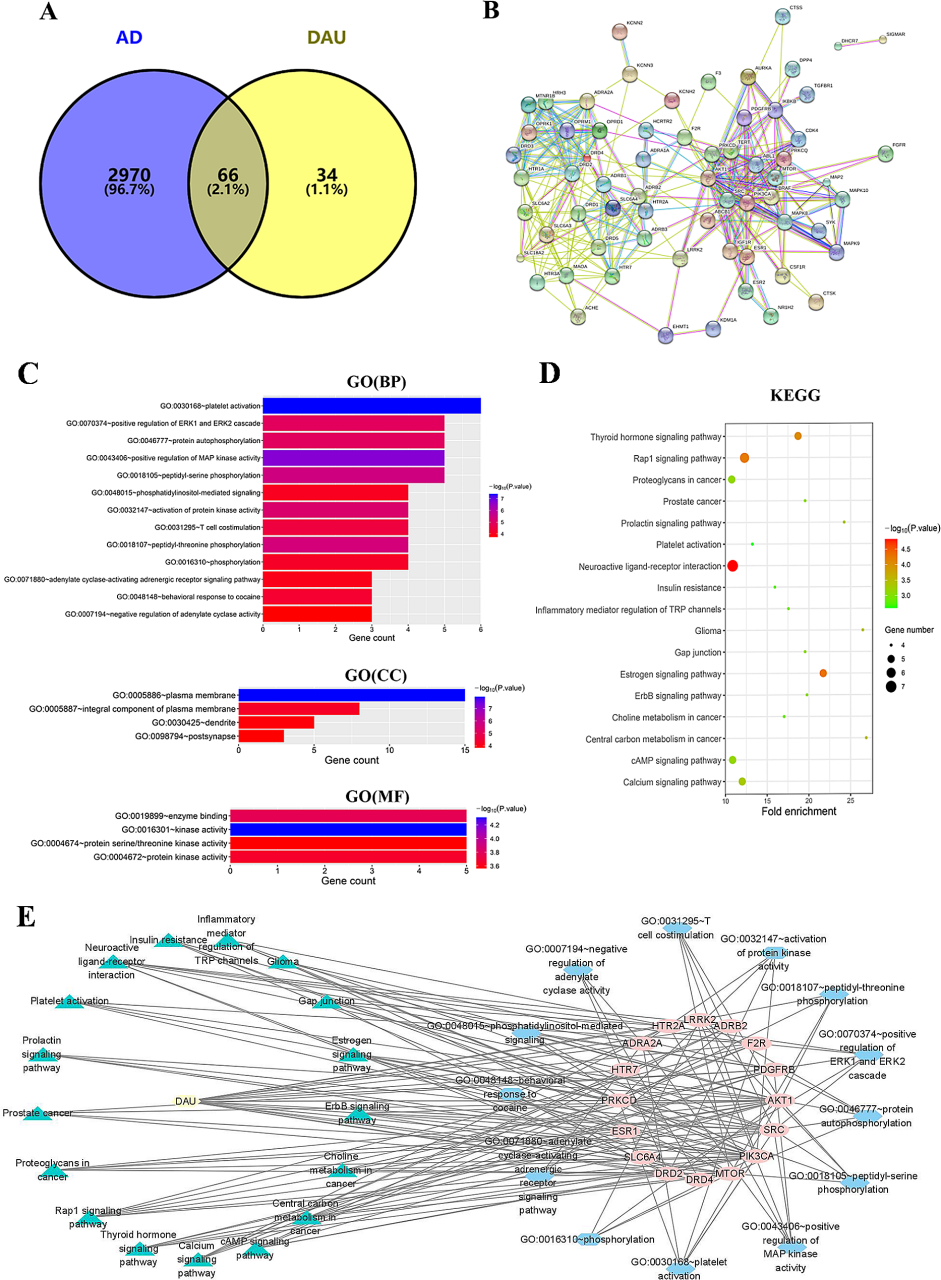
Targets identification and network analysis of DAU for anti-AD activity through network pharmacology. (**A**) Venn diagram depicting common targets of DAU and AD; (**B**) PPI network of core targets. (**C**,** D**) GO function and KEGG pathway enrichment analysis. (**E**) Predicted compound-target-biological process-pathway network

In total, 100 predicted DAU targets associated with drug absorption, distribution, metabolism, and excretion (ADME) were obtained from the SwissADME database, and 3036 AD-related targets from the GeneCards database. Intersecting these datasets yielded 66 common targets (Fig. 1A). PPI network analysis revealed a highly interconnected network with 66 nodes (proteins) and 291 edges (interactions), showcasing an average node degree of 8.82 and a local clustering coefficient of 0.6 (Fig. 1B). Further analysis of STRING-generated protein enrichment tables in Cytoscape 3.8.2 identified 16 core targets from PPI results (Table 2).

**Table 2:** Core targets screened by Cytoscape based on PPI

Serial number	Name	Betweenness centrality	Closeness centrality	Degree
1	AKT1	0.246379123	0.626262626	27
2	SRC	0.180273319	0.596153846	27
3	PIK3CA	0.086604687	0.534482759	21
4	mTOR	0.050750876	0.516666667	20
5	DRD4	0.06829179	0.534482759	18
6	DRD2	0.043133523	0.516666667	15
7	SLC6A4	0.042359017	0.508196721	14
8	ESR1	0.051670003	0.480620155	14
9	PRKCD	0.056078824	0.521008403	13
10	HTR7	0.044799882	0.508196721	13
11	ADRA2A	0.037834557	0.455882353	13
12	HTR2A	0.046998337	0.466165414	12
13	LRRK2	0.033561983	0.484375	8
14	ADRB2	0.018913498	0.480620155	8
15	F2R	0.02559793	0.462686567	8
16	PDGFRB	0.031514182	0.452554745	8

Functional enrichment analysis identified 21 significantly enriched GO terms, including 13 biological processes (BP), 4 cellular components (CC), and 4 molecular functions (MF) terms (Fig. 1C). Prominent enriched BP terms included platelet activation, positive regulation of MAP kinase activity, peptidyl-threonine phosphorylation, protein kinase activity activation, peptidyl-serine phosphorylation, and protein autophosphorylation. Enrichment in CC implicated the plasma membrane, internal component of the plasma membrane, dendrite, and postsynapse. MF terms encompassed kinase activity, enzyme binding, protein kinase activity, and protein serine/threonine kinase activity. These findings suggest DAU primarily targets the cytoplasmic membrane, particularly with relevance to neurosynaptic function, and likely exerts its effects through kinase-level regulation. KEGG pathway enrichment analysis identified 17 potential pathways, involving neuroactive ligand-receptor interaction, estrogen signaling, thyroid hormone signaling, Rap1 signaling and so on (Fig. 1D). According to *P*-value and gene number analysis, the most relevant is the neuroactive ligand-receptor interaction pathway.

Network analysis is used to predict the central target with the best degree of connectivity. Compound-target-biological process-pathway network analysis showed that DAU interacted with multiple potential targets and signaling pathways, highlighting potential synergistic therapeutic effects (Fig. 1E). Based on the degree values in the node table in the network graph, we selected the top ranked targets (Table 3). Considering the relevance of the target and the disease, as well as the pertinent research background, we speculate the most potential mechanism of DAU’s anti-AD effect is related to the regulation of PIK3CA, AKT1 and mTOR.

**Table 3:** Connectivity of core targets from network of “compound-target-biological process-pathway”

Serial number	Name	Degree
1	AKT1	21
2	PIK3CA	20
3	SRC	16
4	mTOR	15
5	PDGFRB	12
6	DRD2	10
7	PRKCD	8
8	HTR2A	8
9	F2R	8
10	ADRA2A	7
11	LRRK2	6
12	ESR1	5
13	ADRB2	5
14	DRD4	4
15	HTR7	3
16	SLC6A4	1

### Molecular docking simulation analysis

**Fig. 2:**
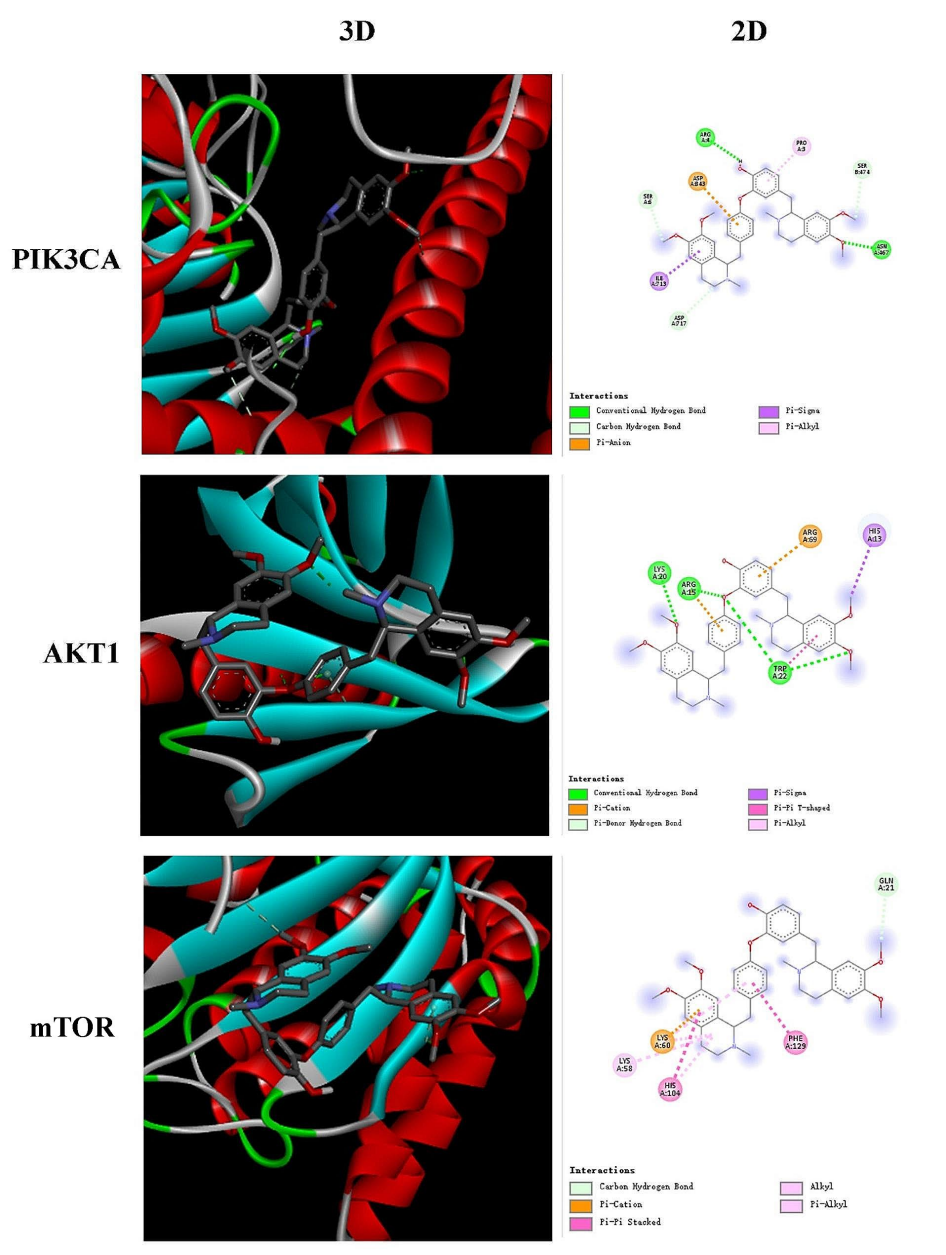
Three-dimensional **(Left)** and two-dimensional **(Right)** docking models of DAU binding to PIK3CA, AKT1 and mTOR

To validate network pharmacology predictions and elucidate the potential binding interactions, molecular docking simulations were performed between DAU (ligand) and the three core targets, PIK3CA, AKT1 and mTOR (receptors). Lower binding energy indicates more stable interaction [26]. DAU exhibited strong binding affinity towards PIK3CA, AKT1 and mTOR, with binding energies of -10.4, -6.8, and − 7.7 kcal/mol, respectively. These findings suggest that these proteins may hold significant therapeutic potential for DAU against AD. Additionally, Fig. 2 visually demostrated the formation of stable DAU-target complexes, further highlighting the reliability of network pharmacology predictions.

### Alleviation of Aβ-induced paralysis by DAU in AD nematodes

To investigate the in vivo anti-AD activity of DAU, AD transgenic nematodes CL4176 and CL2006 were used. These nematodes express human Aβ protein in their body wall muscle cells and exhibit paralysis phenotype at different times as the protein aggregated (CL2006 at 20 °C for ~ 10 days, CL4176 rapidly entered paralysis upon temperature induction). Before the examination, food clearance assay was firstly taken to determine the safe dosing range of DAU in wild type and AD modle nematodes. Results shown in Fig. S1, revealed no influence of DAU treatmen on the growth and development of nematodes by the dose concentration below 80 mM. Considering the concentration of DAU in previous trial studies, we opted for the administration of DAU ≤ 10 µM.

**Fig. 3:**
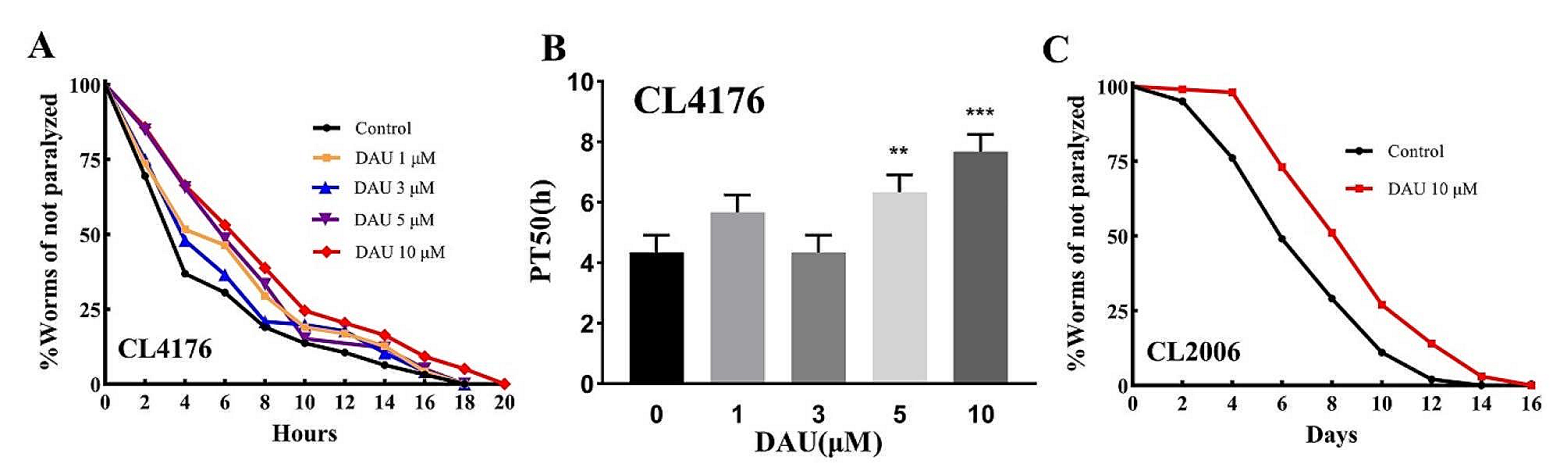
Effect of DAU on Aβ-induced paralysis in CL4176 and CL2006. (**A**) Non-paralysis curves diagram of CL4176; (**B**) The mean of time at which 50% CL4176 worms were paralyzed (PT50) with different doses of DAU. (**C**). Non-paralysis curves diagram of CL2006 with 10 µM DAU. Results are representative of three independent experiments and presented as mean ± SD. *P<0.05

Paralysis assay of CL4176 nematodes were shown in Fig. 3A. DAU treatment significantly shifted the paralysis inhibition curves upwards, and demonstrated an anti-paralytic effect in CL4176 (*P* value shown in Table S1). This observation was supported by the PT50 values (the time to reach half-paralysis) in Fig. 3B, with a paralysis time delay from 4 h (Control) to 6 h (5 µM, *P* = 0.006) and 8 h (10 µM, *P* = 0.0001). The 10 µM DAU group exhibited the longest PT50, demonstrating superior paralysis inhibition efficacy. This optimal concentration was used for subsequent experiments. Furthermore, administration of 10 µM DAU also exhibited a significant anti-paralysis effect in CL2006 (Fig. 3C), reducing the PT50 from 6 h to 10 h (Table S2, *P* < 0.0001), indicating an alleviation of Aβ-induced toxicity. These findings collectively suggest the promising in vivo anti-AD activity of DAU.

### Target-based activation of autophagy by DAU in *C. elegans*

**Fig. 4:**
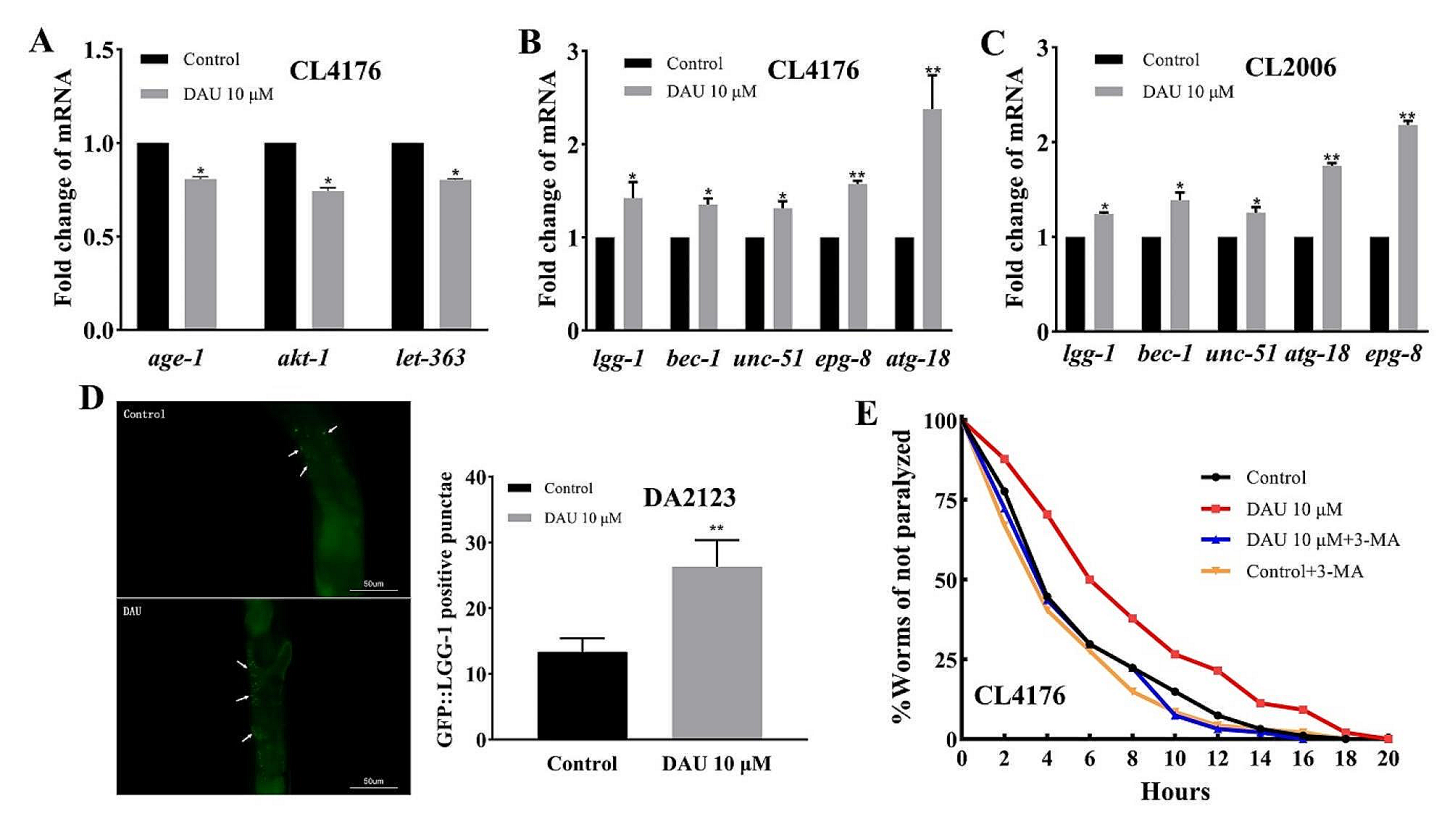
Effect of DAU on autophagy activation. (**A**) Core target genes expression in CL4176 nematodes; (**B**) Autophagy genes expression in CL4176; (**C**) Autophagy genes expression in CL2006; (**D**) Representative micrographs of GFP-positive punctae in DA2123 carrying the *gfp::lgg-1* reporter gene (Left, Scale bar: 50 μm) and quantification of GFP-positive punctae per worm with/without DAU (Right); (**E**) Non-paralysis curves diagram of CL4176 with 3-MA co-treatment. Results are representative of three independent experiments and presented as mean ± SD. * *P* < 0.05; ** *P* < 0.01

In CL4176 nematodes, DAU significantly downregulated the expression levels of *C. elegans* homologs, *age-1* (PIK3CA), *akt-1* (AKT1), and *let-363* (mTOR) (Fig. 4A), validating network pharmacology predictions. Since PI3K/AKT/mTOR signaling pathway regulates autophagy, a key process critical for clearing Aβ aggregates, we investigated DAU’s influence on the expression of autophagy-related genes (*lgg-1*, *bec-1*, *unc-51*, *atg-18*, and *epg-8*) in CL4176 and CL2006 strains. Results shown in Fig. 4B, C revealed that DAU treatment significantly upregulated these genes, indicating potential autophagy activation. This was further supported by the increased GFP-positive punctae (13 to 26) in DA2123 nematodes expressing the *gfp::lgg-1* autophagy reporter gene after DAU administration (Fig. 4D).

To further validate the correlation between autophagy activation and DAU’s anti-AD effect, we co-administered DAU with the autophagy inhibitor 10 µM 3-methyladenine (3-MA, MCE, New Jersey, USA), which primarily inhibits Class PI3K-III activity and prevents autophagosome formation and LC3 expression [27]. Results were shown in Fig. 4E. DAU’s anti-paralysis effect (red curve, *P* = 0.0003) was nullified upon 3-MA co-treatment (blue curve, *P* = 0.3643), while the PT50 were induced from 7 h to 4 h (Table S3), indicating that DAU’s beneficial effect on the inhibition of Aβ-induced paralysis is associated with autophagy activation, particularly autophagosome formation.

### Facilitation of autophagic lysosomal fusion and degradation by DAU in *C. elegans*

**Fig. 5:**
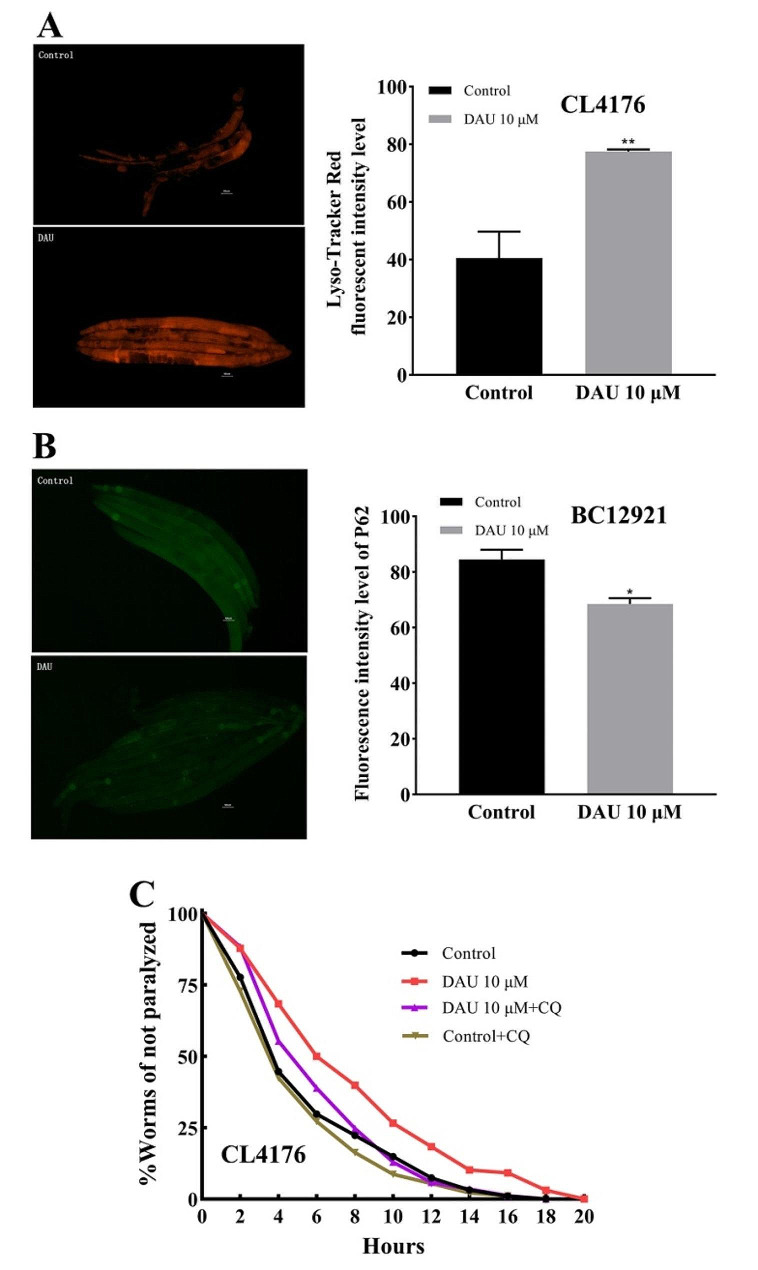
Effect of DAU on autophagic lysosome fusion and degradation in nematodes. (**A**) Lysosome content revealed by Lyso-Tracker red staining in CL4176. Scale bar: 50 μm; (**B**) Expression level of autophagic substrate protein P62::GFP in BC12921. Scale bar: 50 μm; (**C**) Non-paralysis curves diagram of CL4176 co-treated with DAU and CQ. Data represent mean ± SD from three independent experiments. * *P* < 0.05, ** *P* < 0.01

Efficient fusion of autophagosomes with lysosomes and subsequent degradation are crucial for autophagy completion and efficacy. To investigate DAU’s impact on this process, we assessed lysosomal content and autophagic substrate consumption. Lyso-Tracker red dye staining revealed significant increased lysosomal activity in DAU-treated worms compared to the control group (Fig. 5A). Then, we utilized BC12921 strain, expressing GFP-tagged autophagic substrate protein P62, to monitor autophagic substrate consumption. DAU treatment significantly reduced GFP fluorescence intensity (Fig. 5B), indicating increased autophagy degradation activity and autophagic substrate consumption.

To investigate the correlation between DAU’s autophagic degradation activity and its anti-AD efficacy, we co-administered DAU with 100 µM chloroquine (CQ; MCE, New Jersey, USA), an inhibitor of autophagosome-lysosome fusion. DAU’s ameliorative effect (red curve, *P* = 0.0005) against CL4176 paralysis disappeared after co-treatment with CQ (purple curve, *P* = 0.4125) (Fig. 5C, Table S4), indicating that DAU’s anti-AD efficacy was associated with the activation of autophagic lysosomal fusion and degradation.

### Exclusion of autophagy activation by DR in *C. elegans*

**Fig. 6:**
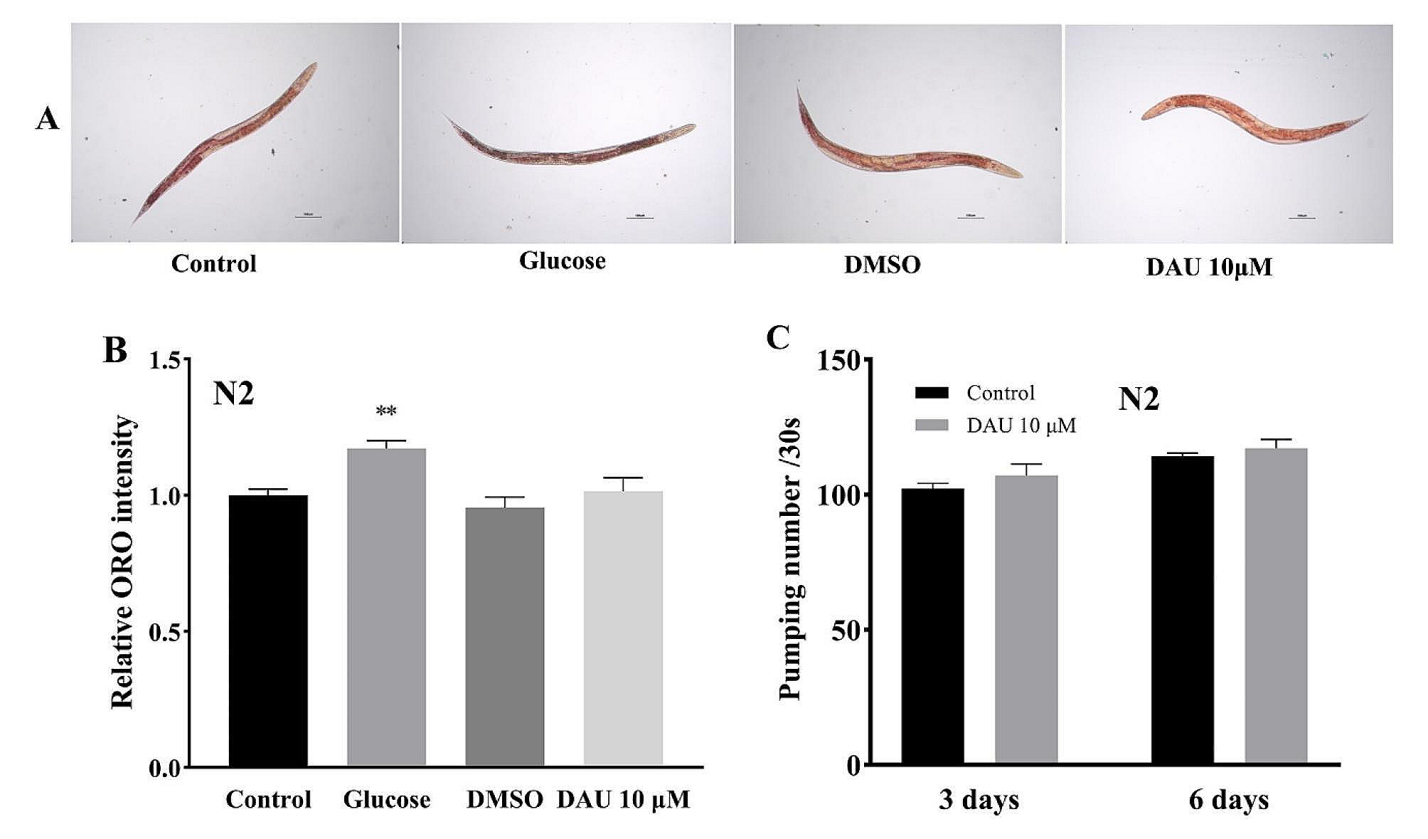
Effect of DAU on the fat accumulation and pharyngeal pump rate in wild type N2 nematodes. (**A**) Representative micrographs of ORO staining of worms treated with Glucose (positive control), DMSO and DUA (Scale bar: 100 μm). (**B**) The quantification of ORO staining intensity per nematode of different groups. (**C**) Pumping number per nematode in 30 s measured on the third and sixth day after DAU treatment. Data are presented as mean ± SD from three independent experiments. ** *P* < 0.01

Dietary restriction (DR) has long been associated with the induction of autophagy and the reduction of proteotoxicity in *C. elegans* and other animals [28]. In order to confirm whether the activation effect of DAU on autophagy is related to DR, we examined the influence of DAU on the fat accumulation and the feeding behavior in *C. elegans* N2. The ORO staining was used to evaluate the effect of DAU on the fat accumulation in nematodes. As shown in Fig. 6A, B, the quantification of staining intensity in glucose-treated worms (high-fat positive control) was significantly higher than control, while the DAU-treated nematodes exhibited no difference with control, indicating no influence of DAU on the lipid content in *C. elegans* and no nutritional deficits exist. Nematode pharyngeal pumping rate was examined to reveal the food intake influenced by DAU, and the results were shown in Fig. 6C. Compared to control, neither day 3 nor day 6 after DAU treatment, there was a significant difference on the pumping number, indicating no effect of DAU on food intake. Therefore, we speculated that the activation effect of DAU on autophagy in nematodes was independed on DR.

### Alleviation of Aβ aggregation by DAU in *C. elegans*

**Fig. 7:**
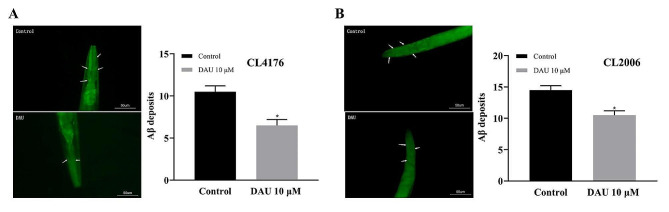
Effect of DAU on Aβ aggregation in CL4176 (**A**) and CL2006 (**B**) strains by Th-T staining. (**Left**) Representative micrographs of Aβ aggregation treated with or without DAU. White arrows indicated Aβ aggregation sites with the scale bar of 50 μm. (**Right**) Statistics on the number of Aβ deposits per nematode in different groups. Error bars represent the mean ± SD. * *P* < 0.05

The pathogenicity of Aβ aggregation stems from the abnormal accumulation of Aβ proteins, and mitigating this aggregation can significantly alleviate the associated toxicity [29]. We investigated the potential correlation between DAU’s anti-AD efficacy and Aβ clearance by assessing Aβ accumulation in CL4176 and CL2006 strains using Th-T staining. Th-T dye exhibits high affinity for Aβ aggregates, enabling quantification of protein aggregation through fluorescence intensity in the anterior pharynx, a key Aβ deposition site in these models. DAU treatment markedly reduced Aβ deposits in the anterior pharyngeal region of both CL4176 and CL2006 strains compared to controls (Fig. 7A, B), suggesting it effectively eliminates abnormal aggregation of Aβ proteins, primarily through autophagy-lysosome pathway.

### Inhibition of Aβ toxicity by DAU at different administration time of *C. elegans*

**Fig. 8:**
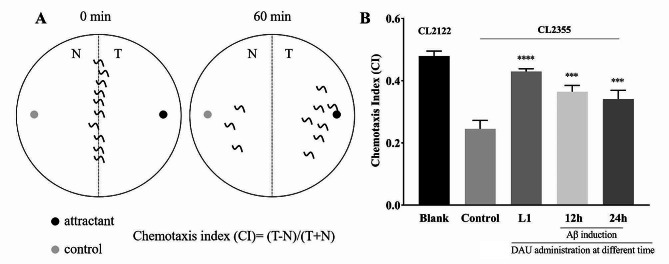
Effect of DAU on Aβ-mediated behavioral dysfunction in CL2355 nematodes. **(A)** Schematic diagram of the chemotaxis assay plate. (**B**) Chemotaxis indexes of CL2355 with DAU administration at different time (L1- before Aβ induction; 12 h/24 h- after Aβ induction). Approximately 300 nematodes were analysed for each treatment and the results are presented as means ± SD of three replicates. *** *P* < 0.001, **** *P* < 0.0001

The impact of drug administration timing on diseases is of significant importance in elucidating the mechanism of drug action and its medicinal application. The aforementioned results indicate that prior treatment of DAU before Aβ induction at L1 larvae holds promising potential as an effective prophylactic medication for AD. In order to explore whether DAU has the same anti-AD effect after the onset of disease, we investigated the neuroprotective capacity of DAU at 12 h/ 24 h after Aβ induction (23 °C) using *C. elegans* CL2355, which contains pan-neuronal Aβ expresion and deficits in chemotaxis behavior [30]. As shown in Fig. 8A, B, compared to the blank strain CL2122 (no Aβ expression), the chemotaxis indexe of CL2355 was decreased from 0.48 to 0.25, demonstrating the neurological impairment in perceiving the attractant benzaldehyde. After DAU administration, no matter when the drug treated before (L1 larvae) or after Aβ induction (12 h/ 24 h), the chemotaxis indexes of DAU groups were significantly increased to 0.43, 0.36 and 0.34 compared to the untreated CL2355. This result confirmed the protective effect of DAU at older ages of AD nematodes, indicating a persistent benefits of autophagy activation to potential therapeutic efficacy of DAU.

### Clearance of polyQ proteins by DAU in *C. elegans*

**Fig. 9:**
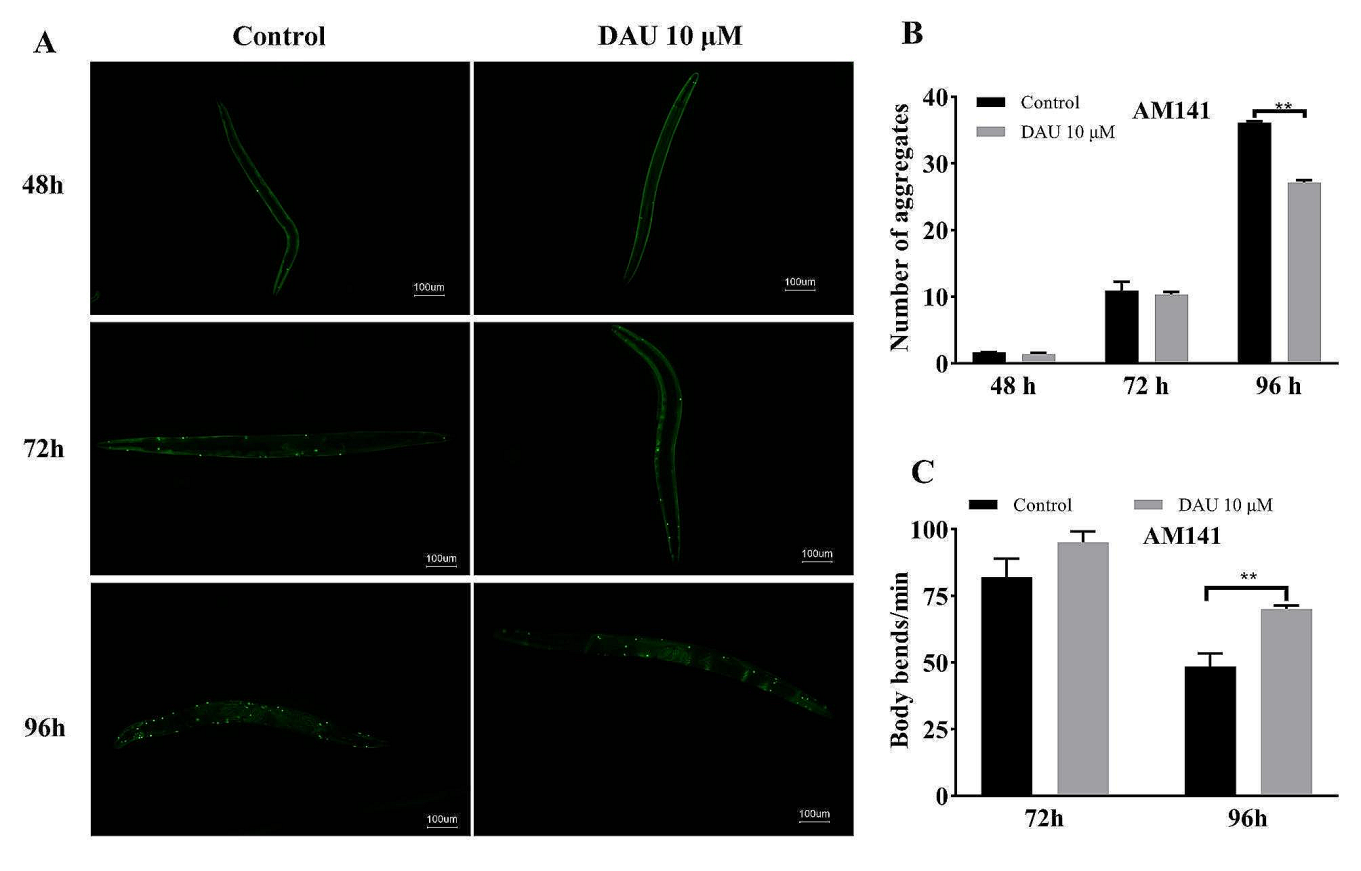
Effect of DAU on PolyQ aggregation and motility in AM141 nematodes. (**A**) Fluorescence microscopy images of AM141 nematodes at 48, 72, and 96 h post DAU treatment. Scale bars: 100 μm. (**B**) Quantification of polyQ40::YFP aggregates. (**C**) Body bends number of AM141 per min. Data present mean ± SD from three independent experiments. ** *P* < 0.01

Pathogenic protein aggregation is a major molecular mechanism in protein conformational diseases, including AD and HD. We explored DAU’s potential to scavenge polyglutamine (polyQ), the pathogenic protein of HD, using a HD model strain AM141. This strain expresses polyQ40::YFP fusion proteins in body-wall muscle cells, resulting in discrete fluorescence aggregates over time (Fig. 9A) and malfunction of motility. Results were shown in Fig. 9. Although early DAU treatment (48 h, 72 h) didn’t significantly impact polyQ aggregation and toxicity, it markedly reduced the number of fluorescent aggregates in AM141 body wall (Fig. 9B) and improved the rate of body bends at 96 h post-DAU administration (Fig. 9C). These findings suggest that DAU can reduce toxic protein PolyQ aggregation, which should be also related to the activation of autophagy-lysosome protein degradation pathway by DAU.

## Discussion

This study employed network pharmacology to identify 66 potential DAU targets against AD. PPI network analysis pinpointed 16 core targets. PIK3CA, AKT1 and mTOR were predicted to be the central targets with the best connectivity through the analysis of “compound-target-biological process-pathway network”. GO enrichment analysis indicated DAU’s anti-AD effects primarily occur in the cytoplasmic membrane and synaptic links through regulating protein kinase or phosphorylation levels. KEGG pathway analysis revealed neuroactive ligand-receptor interactions as the primary pathway involved. Studies have shown that neuroactive ligands affect neuronal function by binding to intracellular receptors, which can bind to transcription factors and regulate gene expression. Disruption of genes in this pathway can lead to decreased memory function [31].

PIK3CA and AKT1 are crucial kinases in the PI3K/AKT signaling pathway, which regulates multiple pathways for AD treatment, including reducing inflammatory factors’ expression levels in AD mice by regulating downstream FoxO3a, improving hyperphosphorylation of tau in AD rats, and promoting proliferation of AD model cells by regulating downstream GSK-3β [32, 33]. In our in vivo study, 10 µM DAU effectively delayed paralysis in AD nematodes, suggesting its anti-AD potential. Further supporting this, DAU significantly downregulated the expression of PI3K, AKT, and mTOR homologs *age-1*, *akt-1*, and *let-363* in nematodes. Previous studies have shown that inhibition of PI3K, AKT, and mTOR can block the neuronal cycle events induced by Aβ oligomers [34]. The PI3K/AKT/mTOR pathway negatively regulates autophagy [35]. Studies have shown that DAU can inhibit the PI3K/AKT/mTOR signaling pathway, inducing autophagic apoptosis in cancer cells by mediating ROS generation [36]. This pathway is involved in AD pathogenesis by affecting synaptic plasticity, neuronal polarization, neurotransmitter release, and protein homeostasis [35, 37]. Persistent activation of this pathway is an early pathological feature in AD patients, making its inhibition an effective therapeutic strategy for AD. Graphene oxide, for example, improved learning and memory impairment in AD mice through inhibiting PI3K/AKT/mTOR pathway to induce autophagy [38].

Autophagy is a crucial self-protective mechanism that maintains intracellular homeostasis through the cooperation of autophagic vesicles and lysosomes to degrade abnormal organelles or substances such as proteins [39]. The process can be initiated by inhibiting PI3K/AKT/mTOR, which activates the ULK1 complex to phosphorylate Bec-1 and forms phagocytic vesicles with a free bilayer membrane structure. Bec-1 functions as a marker protein for the initiation of autophagy synthesis complexes, while ATG-14 facilitates phagocytic vesicle formation and nucleation [40]. Additionally, LC3 (LGG-1 in nematodes) and ATG-18 assist in extending the autophagic membranes and forming autophagic vesicles, which subsequently bind to lysosomes to form autophagy-lysosomes. P62 acts as an autophagy-specific substrate binding protein that is degraded by degradative enzymes within lysosomes along with its substrate [41]. Our study demonstrated that DAU upregulated the expression of autophagy-related genes and the autophagosome marker protein LGG-1, increased lysosomal content, and enhanced autophagolysosome fusion and degradation activities, which effectively eliminated Aβ deposition in AD nematodes. Co-treatment with autophagy inhibitors 3-MA and CQ revealed that the activation of autophagy was essential for DAU’s anti-AD activity. These findings indicate DAU’s ability to significantly reduce Aβ protein aggregation and toxicity, restore protein homeostasis, and exert anti-AD effects through the autophagy-lysosomal protein clearance pathway.

Further experiments showed that DAU treatment inhibited abnormal polyQ protein aggregation in HD nematodes, indicating its potential to enhance the clearance of heteropoly proteins via the autophagy-lysosomal pathway and restore bioactivity for treating other neurodegenerative diseases. Studies have demonstrated that the extract of Folium *Hibisci Mutabilis* exhibits potent autophagy-enhancing properties, resulting in a significant augmentation of GFP::LGG-1 positive sites in DA2123 [42]. Moreover, it effectively eliminates aberrant aggregates of Aβ, α-synuclein, and polyQ40 in *C. elegans*, thereby extending lifespan and ameliorating behavioral deficits in nematodes. In addition to the nematode model, activation of autophagy has also shown efficacy in clearing polyQ aggregates in other cell and Drosophila models [43].

## Conclusion

Based on network pharmacology and molecular docking simulations, PIK3CA, AKT1, and mTOR were indentified as the core targets involved in DAU’s anti-AD activity. In *C. elegans* models of AD, DAU effectively reduced Aβ-induced paralysis, indicating its functional anti-AD activity. This therapeutic effect can be attributed to the activation of Aβ clearance via the autophagy-lysosomal pathway. Furthermore, DAU also demonstrated efficacy in reducing PolyQ protein aggregation, suggesting its potential application in treating other neurodegenerative diseases characterized by protein aggregation. These findings provide data support for further exploration of DAU’s therapeutic potential not only for AD but also for other protein aggregation-related neurodegenerative diseases.

### Electronic supplementary material

Below is the link to the electronic supplementary material.

**Supplementary Material 1**: The original data of Network pharmacology analysis

**Supplementary Material 2**: Fig. S1: Food clearance assay of DAU at different concentrations in CL2006 and N2 nematode. Table S1: Effect of DAU on Aβ-induced paralysis in CL4176. Table S2: Effect of DAU on Aβ-induced paralysis in CL2006. Table S3: Effect of co-treatment of DAU and inhibitor 3-MA on Aβ-induced paralysis in CL4176. Table S4: Effect of co-treatment of DAU and inhibitor CQ on Aβ-induced paralysis in CL4176

## Data Availability

The raw data for the network pharmacological analysis can be viewed in the supplementary file. The results of “Food clearance assay” and tables with respective *P* values of each paralysis data were added in the supplementary file too. The other relevant figures and tables are all included in the text.

## Abbreviations

AD: Alzheimer’s disease
DAU: Dauricine
Aβ: Amyloid beta
*C. elegans*: *Caenorhabditis elegans*
*E. coli*: *Escherichia coli*
FuDR: 5-fluoro-2’-deoxyuridine
NGM: Nematode growth medium
HD: Huntington’s disease
GO: Gene ontology
KEGG: Kyoto encyclopedia of genes and genomes
Th-T: Thioflavin T
CQ: Chloroquine
3-MA: 3-Methyladenine
