# Supplementary material for: Network pharmacology-based mechanism analysis of dauricine on the alleviating Aβ-induced neurotoxicity in Caenorhabditis elegans
Source: BMC Complement Med Ther. 2024 Aug 30;24:321. doi: 10.1186/s12906-024-04589-w (PMC11363685; doi:10.1186/s12906-024-04589-w)
Supplement: Supplementary file 2 — Supplementary Material 2: Fig. S1: Food clearance assay of DAU at different concentrations in CL2006 and N2 nematode. Table S1: Effect of DAU on Aβ-induced paralysis in CL4176. Table S2: Effect of DAU on Aβ-induced paralysis in CL2006. Table S3: Effect of co-treatment of DAU and inhibitor 3-MA on Aβ-induced paralysis in CL4176. Table S4: Effect of co-treatment of DAU and inhibitor CQ on Aβ-induced paralysis in CL4176 [file 12906_2024_4589_MOESM2_ESM.docx]

**Supplementary File 2:**


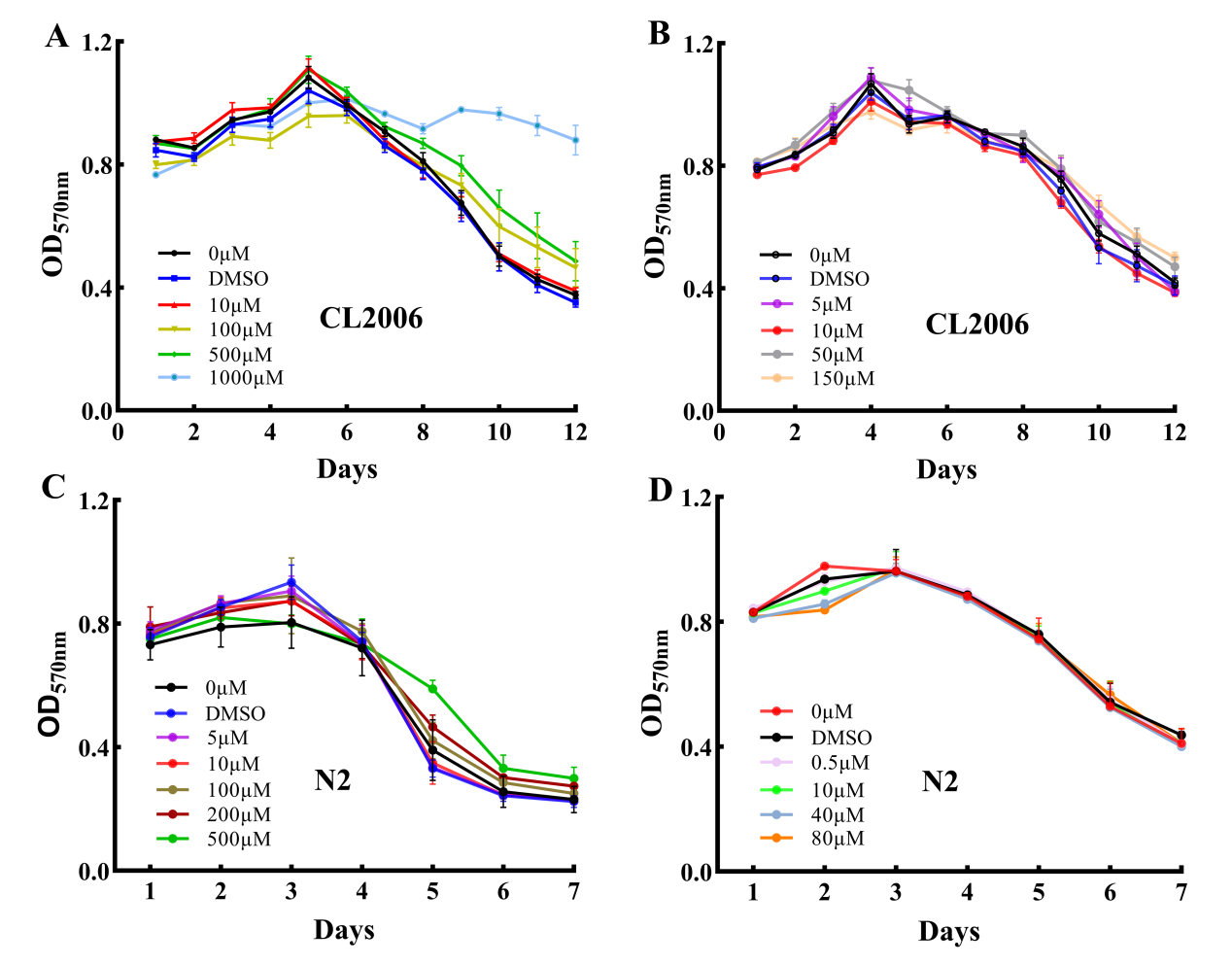


**Fig. S1:** Food clearance assay of DAU at different concentrations in CL2006 (A,B) and N2 (C,D) nematodes. L1 larvae worms were cultured at 20℃ in 96 well plates with or without DAU (10-15 worms per well, 10 wells replicates) at different concentrations. The initial value of OD570 nm of NA22 was ≈0.80, and *E. coli* reduction rate was measured daily for 7 days using a full-wavelength microplate reader.

**Table S1:** Effect of DAU on Aβ-induced paralysis in CL4176

| **Groups** | **PT50 (h)** | ***P* value** |
| --- | --- | --- |
| Control | 4 |  |
| DAU 1 μM | 6 | 0.0760 |
| DAU 3 μM | 4 | 0.2071 |
| DAU 5 μM | 6 | 0.0071 |
| DAU 10 μM | 8 | 0.0003 |

**Table S2:** Effect of DAU on Aβ-induced paralysis in CL2006

| **Groups** | **PT50 (h)** | ***P* value** |
| --- | --- | --- |
| Control | 6 |  |
| DAU 10 μM | 10 | <0.0001 |

**Table S3:** Effect of co-treatment of DAU and inhibitor 3-MA on Aβ-induced paralysis in CL4176

| **Groups** | **PT50 (h)** | ***P* value** |
| --- | --- | --- |
| Control | 4 |  |
| DAU 10 μM | 7 | 0.0003 |
| DAU 10 μM+3-MA | 4 | 0.3643 |
| Control+3-MA | 4 | 0.3029 |

**Table S4:** Effect of co-treatment of DAU and inhibitor CQ on Aβ-induced paralysis in CL4176

| **Groups** | **PT50 (h)** | ***P* value** |
| --- | --- | --- |
| Control | 4 |  |
| DAU 10 μM | 7 | 0.0005 |
| DAU 10 μM+CQ | 6 | 0.4125 |
| Control+CQ | 4 | 0.3641 |
